# Association between Serum 25-Hydroxyvitamin D and Inflammatory Cytokines in Healthy Adults

**DOI:** 10.3390/nu6010221

**Published:** 2014-01-02

**Authors:** Xiaomin Sun, Zhen-Bo Cao, Yuping Zhang, Yoshiko Ishimi, Izumi Tabata, Mitsuru Higuchi

**Affiliations:** 1 Graduate School of Sport Sciences, Waseda University, Tokorozawa, Saitama 359-1192, Japan; E-Mails: gzhtxiaomin@ruri.waseda.jp (X.S.); rockets912@hotmail.com (Y.Z.); 2 Faculty of Sport Sciences, Waseda University, Tokorozawa, Saitama 359-1192, Japan; E-Mail: mhiguchi@waseda.jp; 3 National Institute of Health and Nutrition, Tokyo 162-8636, Japan; E-Mail: ishimi@nih.go.jp; 4 Faculty of Sport and Health Science, Ritsumeikan University, Kusatsu, Shiga 525-8577, Japan; E-Mail: tabatai@fc.ritsumei.ac.jp

**Keywords:** vitamin D, interferon-γ, interleukin-6, interleukin-17, physical activity

## Abstract

Here, we aimed to examine the associations between levels of serum 25-hydroxyvitamin D [25(OH)D] and inflammatory cytokines in healthy Japanese adults. A total of 95 healthy adults (61 women; age range 21–69 years) participated in our study. Fasting blood samples were analyzed for 25(OH)D, 1,25-dihydroxyvitamin D [1,25(OH)_2_D], interferon-γ (IFN-γ), interleukin-6 (IL-6), and interleukin-17 (IL-17) levels using enzyme-linked immunosorbent assays kits. Total percent body fat was determined by dual energy X-ray absorptiometry (DXA). Moderate to vigorous physical activity (MVPA) was assessed objectively using an activity monitor for 7 days. The mean 25(OH)D concentration was 34.7 nmol/L, and 83 subjects had 25(OH)D concentrations less than 50 nmol/L. Multiple linear regression analysis revealed that serum 25(OH)D level was positively related to plasma IL-17 level (β = 0.26, *p* = 0.025), after adjustment for gender, age, vitamin D intake, alcohol consumption, smoking status, and percent body fat. This relationship remained statistically significant (β = 0.28, *p* = 0.019) even after additional adjustment for MVPA. However, no significant association was found between serum 25(OH)D level and plasma IFN-γ or IL-6 levels. In conclusion, this study identified a high prevalence of vitamin D deficiency in healthy Japanese adults. Serum 25(OH)D level was positively related to IL-17 level, independent of physical activity.

## 1. Introduction

Vitamin D is of vital importance for bone health and also appears to have extra-skeletal effects. A low level of 25-hydroxyvitamin D [25(OH)D], the storage form of vitamin D and the form used in the evaluation of vitamin D status, has been associated with diabetes, cardiovascular diseases, and cancers [1,2]. Among other non-classic vitamin D effects, the regulation of the immune system has received increasing attention in the past few years.

Vitamin D regulates inflammatory cytokines, which contribute to immune signaling and host defenses. *In* *vitro* studies have found that vitamin D can inhibit the production of the proinflammatory cytokines interleukin-17 (IL-17), interferon-γ (IFN-γ), and interleukin-6 (IL-6) [3,4,5]. Animal studies have also found that vitamin D reduces the production of IL-6, IFN-γ, and tumor necrosis factor-α (TNF-α) [6,7,8]. However, human studies investigating the relationships between vitamin D and inflammatory cytokines are scarce and have yielded inconsistent results, especially in healthy adults [9,10]. Barker *et al.* [9] found that serum 25(OH)D concentrations were inversely correlated with IFN-γ levels, but not with TNF-α or IL-10 levels, in healthy young women (*n* = 12) and men (*n* = 16). Conversely, Peterson *et al.* [10] reported that serum 25(OH)D concentrations are negatively correlated with serum TNF-α levels, but not with IL-6 or IL-10 levels, in healthy women (*n* = 69). Those studies focused on IFN-γ, IL-10, IL-6, and TNF-α. IL-17 is a recently discovered cytokine produced primarily in T-helper 17 cells. IL-17 plays an important role in immunity, not only in inducing and maintaining chronic inflammatory diseases, but also in providing protection against infection [11]. However, the relationship between serum 25(OH)D and IL-17 levels in healthy adults is not well understood. Moreover, previous studies did not consider the confounding effects of physical activity. A number of studies have shown that higher physical activity levels are associated with lower circulating levels of proinflammatory cytokines and higher levels of serum 25(OH)D [12,13,14].

Thus, the purpose of this study was to evaluate the association between serum 25(OH)D and inflammatory cytokines (IFN-γ, IL-6, and IL-17) in healthy Japanese adults. We also investigated whether these associations are independent of physical activity, quantified using objective measures of moderate to vigorous physical activity (MVPA).

## 2. Experimental Section

### 2.1. Subjects

A total of 95 healthy Japanese adults aged from 21 to 69 years (61 women) were recruited through the internet, a poster, and a local newspaper insert in Saitama, Japan (35° N latitude). None of the subjects had been diagnosed with cardiac disease or chronic renal failure, and none were taking any medications that could affect the study variables (*i.e.*, vitamin D supplements, vitamin D analogues, calcium, or any drugs that could affect bone and mineral metabolism, including bisphosphonates). The purpose, procedures, and risks of the study were explained to each subject prior to inclusion, and all subjects gave written informed consent before participating. All procedures were reviewed and approved by the Ethical Committee of Waseda University.

### 2.2. Body Composition

Height (without shoes) was measured to the nearest millimeter using a stadiometer (YL-65, Yagami Inc., Nagoya, Japan). Body mass was measured using an electronic scale with the subjects wearing light clothing and no shoes, and was determined to the nearest 0.1 kg (Inner Scan BC-660, Tanita Co., Tokyo, Japan). Body mass index (BMI) was calculated by dividing the body mass in kilograms by the square of height in meters (kg/m^2^). Percent body fat was measured using dual-energy X-ray absorptiometry (DXA) (Hologic QDT-4500, DXA Scanner, Hologic Inc., Whaltham, MA, USA).

### 2.3. Blood Sampling

Venous blood samples were collected after 12-h overnight fasting in early spring. Serum and plasma samples were stored at −80 °C until subsequent analyses. Serum 25(OH)D, 1,25(OH)_2_D, intact parathyroid hormone (iPTH), and plasma inflammatory cytokine (IFN-γ, IL-6, and IL-17) concentrations were measured using commercially available enzyme-linked immunosorbent assay (ELISA) kits [25(OH)D and 1,25(OH)_2_D: Immundiagnostik AG, Bensheim, Germany; iPTH: Abnova, Jhongli, Taiwan; IFN-γ: BD Biosciences, San Diego, CA, USA; IL-6: HS600B, R & D Systems, Minneapolis, MN, USA; IL-17: Diaclone SAS, Besancon, France]. The intra-assay and inter-assay coefficients of variation were 8.9% and 10.6% for serum 25(OH)D, 6.2% and 6.0% for 1,25(OH)_2_D, 3.5% and 5.2% for iPTH, 4.2% and 12.8% for IFN-γ, 7.3% and 7.8% for IL-6, and 11.1% and 11.8% for IL-17.

### 2.4. Lifestyle Variables (Physical Activity, Vitamin D Intake, Alcohol Consumption, and Smoking Status)

Physical activity (PA) was assessed objectively using an activity monitor (Kenz Lifecorder Plus, Suzuken Co. Ltd., Nagoya, Japan) worn continuously for 10 days. Subjects with fewer than 7 days (5 weekdays and 2 weekend days) of activity recorded were eliminated from the data analysis (2 adults). All participants were instructed in the instrument’s use, and wore it on their belt or waistband from the moment they got up until they went to bed, except while bathing or swimming. MVPA from the activity monitor was determined using 7 days of monitored activity, according to the activity intensity levels defined by the manufacturer’s analysis program. Additional details have been published elsewhere [15,16]. Information on vitamin D intake was assessed by a brief self-administered diet history questionnaire (BDHQ) [17,18]. Smoking status and alcohol consumption were obtained using a questionnaire. Smoking status was classified as never, past, or current smokers, and alcohol consumption status was classified as nondrinkers or current drinkers who averaged 0.1–22.9, 23.0–45.9, 46.0–68.9, or ≥69.0 g alcohol/day [19].

### 2.5. Statistical Analysis

Descriptive statistics for all variables are expressed as mean ± SD. Data were checked for normality with a Kolmogorov-Smirnov test prior to all statistical analyses. When normal distribution could not be accepted, variables were logarithmic-transformed or reciprocal-of-square-root transformed before statistical analysis. Student’s *t* tests were used to determine gender differences in subject characteristics and measured outcomes. Pearson’s correlation coefficients were computed among serum 25(OH)D, 1,25(OH)_2_D, iPTH, and plasma inflammatory cytokines (IFN-γ, IL-6, IL-17). Multiple linear regression analyses were performed to assess the associations between serum 25(OH)D levels and levels of plasma inflammatory cytokines, adjusted for gender, age, vitamin D intake, alcohol consumption and smoking status, percent body fat, and further adjusted for MVPA. Statistical analyses were performed using IBM SPSS Statistics 20 for Windows (SPSS Inc., Chicago, IL, USA). The statistical significance level was set at *p* < 0.05.

## 3. Results

Subject characteristics and blood parameters according to gender are presented in Table 1. Height, weight, BMI, and 25(OH)D levels were significantly higher in men than in women (*p* < 0.001), while percent body fat levels were significantly lower in men than in women (*p* < 0.001). Furthermore, no significant differences were found in age, 1,25(OH)_2_D, plasma inflammatory cytokines, iPTH, MVPA, or vitamin D intake between men and women. In the present study, the average serum 25(OH)D concentration was 34.7 nmol/L. The overall prevalence rates of vitamin D sufficiency (≥75 nmol/L), insufficiency (50 to 75 nmol/L), and deficiency (<50 nmol/L) were 1%, 12%, and 87%, respectively. In the present study, 19.6% of our subjects drank more than 46.0 g/day of alcohol, and 30.1% were current or past smokers.

**Table 1:** Subject characteristics according to gender.

Variable	Total (*n* = 95)	Men (*n* = 34)	Women (*n* = 61)
Age (years)	44 ± 14	42 ± 16	44 ± 13
Height (cm) ^#^	162.5 ± 7.9	170.3 ± 5.9	158.1 ± 4.9
Weight (kg)^ #^	58.7 ± 10.4	69.0 ± 6.4	53.0 ± 7.3
BMI (kg/m^2^) ^#^	22.1 ± 3.0	23.9 ± 2.6	21.2 ± 2.8
Body fat (%) ^#^	23.4 ± 6.4	17.9 ± 4.6	26.5 ± 5.0
25(OH)D (nmol/L) ^#^	34.7 ± 16.4	42.1 ± 20.2	30.6 ± 12.1
1,25(OH)_2_D (pg/mL)	38.9 ± 9.8	40.8 ± 10.1	37.8 ± 9.6
IL-6 (pg/mL)	0.47 ± 0.53	0.42 ± 0.42	0.49 ± 0.58
IFN-γ (pg/mL)	1.14 ± 1.34	1.49 ± 1.86	0.95 ± 0.89
IL-17 (pg/mL)	21.7 ± 30.1	23.3 ± 32.7	20.9 ± 28.8
iPTH (pg/mL)	63.1 ± 21.6	58.1 ± 21.2	66.0 ± 21.5
MVPA (min/day) ^†^	33.7 ± 21.1	34.7 ± 23.6	33.1 ± 19.8
Vitamin D intake (μg/day)	6.5 ± 4.2	5.3 ± 2.4	7.1 ± 4.9

Data are expressed as means ± SD. BMI, body mass index; 25(OH)D, 25-hydroxyvitamin D; 1,25(OH)_2_D, 1,25-dihydroxyvitamin D; IL, interleukin; IFN, interferon; iPTH, intact parathyroid hormone; MVPA, moderate to vigorous physical activity; ^#^ Significant difference between men and women, *p* < 0.001; ^†^ *n* = 93.

**Figure 1:**
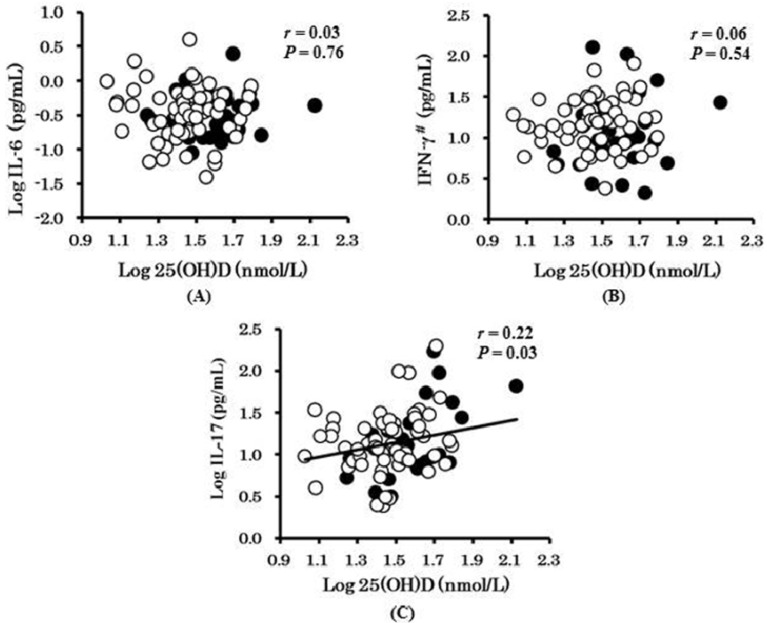
The relationship between serum 25(OH)D and IL-6 (**A**); IFN-γ (**B**); and IL-17 (**C**) in healthy Japanese adults. Data were logarithmic-transformed or ^#^ reciprocal-of-square-root transformed before performing the analysis. Open and closed circles represent data from women and men, respectively.

Figure 1 shows the relationships between serum 25(OH)D concentrations and levels of inflammatory cytokines (IFN-γ, IL-6, and IL-17). Serum 25(OH)D concentrations were positively correlated with 1,25(OH)_2_D levels (*r* = 0.34, *p* = 0.001) and plasma IL-17 levels (*r* = 0.22, *p* = 0.031), but not with IFN-γ, IL-6, or iPTH levels. Furthermore, IFN-γ, IL-6, IL-17, and iPTH concentrations were not significantly associated with the concentrations of 1,25(OH)_2_D (data not shown).

In order to investigate whether serum 25(OH)D levels were independently related to IL-17 levels, multivariate linear regression analyses using IL-17 as the dependent variable were performed (Table 2). As shown in Models 1 and 2, 25(OH)D levels were positively related to IL-17 levels after adjustment for gender and age (β = 0.29, *p* = 0.010), and after further adjustment for vitamin D intake, alcohol consumption, smoking status, and percent body fat (β = 0.26, *p* = 0.025). This relationship remained statistically significant (β = 0.28, *p* = 0.019) after additional adjustment for MVPA (Model 3).

**Table 2:** Results from multiple linear regression analyses examining the association between serum 25(OH)D and plasma IL-17.

Log IL-17	B	β	*P*
**Model 1**			
Log serum 25(OH)D (nmol/L)	0.592	0.288	0.010
**Model 2**			
Log serum 25(OH)D (nmol/L)	0.543	0.264	0.025
**Model 3**			
Log serum 25(OH)D (nmol/L)	0.579	0.282	0.019

25(OH)D, 25-hydroxyvitamin D; IL, interleukin; B, unstandardised regression coefficients; β, standardised regression coefficients. IL-17 and serum 25(OH)D were log-transformed before performing the analysis. Model 1 was adjusted for gender and age; Model 2 included model 1 plus adjustment for vitamin D intake, alcohol consumption, smoking status, and percent body fat; Model 3 included model 2 plus adjustment for MVPA.

## 4. Discussion

This cross-sectional study was performed to examine whether serum 25(OH)D levels are associated with inflammatory cytokine levels in healthy Japanese adults aged 21–69 years. There was a high prevalence of serum 25(OH)D deficiency in our study, consistent with the results of other studies in Japanese subjects [20,21,22,23,24], which have mainly focused on women and the elderly. Furthermore, this study showed that serum 25(OH)D levels were not significantly correlated with plasma IFN-γ or IL-6 levels, but were positively correlated with plasma IL-17 levels in healthy Japanese adults. This positive relationship between serum 25(OH)D and IL-17 levels remained significant even after controlling for potential confounding factors such as gender, age, vitamin D intake, alcohol consumption, smoking status, percent body fat, and MVPA.

Serum 25(OH)D levels are mainly influenced by sunlight exposure and vitamin D intake from foods and supplements [25,26]. Participants in the present study lived at a high northern latitude (35°), where vitamin D synthesis in the skin is absent during winter and early spring [25,27]. We previously found that the average vitamin D intake is 6.5 μg/day, which is higher than the recommended adequate intake (5.5 μg/day) for Japanese [28]. Despite this higher daily vitamin D intake, as many as 87% of subjects in the present study had serum 25(OH)D concentrations less than 50 nmol/L, which is considered deficient. This suggests that subjects who live in higher latitudes may need to consume more vitamin D from foods or supplements, and at least more than 5.5 μg/day during the Japanese winter and early spring.

Several studies have examined the influence of vitamin D on levels of inflammatory cytokines in healthy adults, but the results have been inconsistent. Peterson *et al.* [10] have reported that serum 25(OH)D levels were negatively correlated with serum TNF-α levels, but were not correlated with IL-6 levels, in healthy women over a broad age range. However, Barker *et al.* [9] have reported that serum 25(OH)D levels had a significantly negative relationship with IFN-γ levels, but not with TNF-α levels. In that study, the relationship between serum 25(OH)D and IFN-γ was investigated in only 28 adults, and none of the potential confounding variables such as age, gender, and vitamin D intake were considered. Furthermore, neither of these previous studies of healthy adults investigated the confounding effect of MVPA, measured objectively, on the relationship between serum 25(OH)D and inflammatory cytokines. Panagiotakos *et al.* [12] and Scragg *et al.* [14] have demonstrated that a high degree of physical activity is associated with attenuated circulating levels of inflammatory cytokine and highly active individuals have significantly increased circulating levels of 25(OH)D compared with inactive individuals. Therefore, the confounding effect of PA needs to be considered when investigating the association between serum 25(OH)D and inflammatory cytokines [12,13,14]. In the present study, serum 25(OH)D concentrations were not significantly related to IL-6 or IFN-γ levels, even after adjustment for gender, age, vitamin D intake, alcohol consumption, smoking status, percent body fat, and MVPA. A potential explanation for the discrepancy between our study and the above mentioned reports may be related to differences in the prevalence of vitamin D deficiency (25(OH)D < 50 nmol/L), which is higher in our subjects (87% *versus* <15%).

A novel finding of our investigation was the significantly positive relationship between serum 25(OH)D and IL-17 concentrations, even after adjustment for several covariates. However, this result is potentially in conflict with results in previous studies in patients with multiple sclerosis and asthma, which have shown a negative relationship between vitamin D and IL-17 [4,29]. Recently, IL-17 was found to be secreted not only from T cells, but also from many other cell types, including epithelial cells, keratinocytes, natural killer cells, and macrophages [11,30]. IL-17 plays an important role in providing protection against infection and in inducing and maintaining chronic inflammatory diseases [11].

In many autoimmune diseases, IL-17 mediates adverse effects by its induction of pro-inflammatory cytokines, which contribute to the establishment of a chronic inflammatory state [30]. Conversely, neutralization of IL-17 could completely block the induction of genes encoding antimicrobial peptides, which provide protection from infection [31]. This suggests that higher levels of IL-17, especially when secreted from innate immunity cells, may play an important role in host defense in humans. On the other hand, 1,25(OH)_2_D is a direct regulator of antimicrobial innate immune responses in isolated human keratinocytes, monocytes, and other cell types. In African Americans, lower serum 25(OH)D levels were found to be inefficient in supporting cathelicidin messenger RNA induction [32]. Furthermore, serum 25(OH)D levels have been found to be positively correlated with plasma antimicrobial peptide levels in healthy adults with low 25(OH)D concentrations (≤80 nmol/L) [33,34]. According to these data, elevated serum 25(OH)D concentrations were positively associated with increased IL-17 concentrations in healthy subjects with low serum levels of 25(OH)D, which is probably related to innate immunological function. However, this relationship has not been investigated in humans with higher levels of 25(OH)D. Although Peric *et al.* [35] have found that 1,25(OH)_2_D probably enables IL-17 to increase cathelicidin in keratinocytes, the mechanism is still not well understood.

The present study has several limitations. First, this study used a cross-sectional design, rather than a longitudinal design, and therefore it cannot provide causal evidence on the association between serum vitamin D and inflammatory cytokine levels. Second, this study included only healthy Japanese adults, and thus it is unknown whether the same associations exist in people of other ethnicities, because vitamin D metabolism and circulating 25(OH)D concentrations vary substantially by race [36].

An understanding of this issue in other populations is needed. Third, although we took into account the confounding effect of gender by applying multiple linear regression, because men comprised just a small proportion of our subjects, our results should be interpreted with caution and confirmed in larger samples of men. Fourth, because of the high prevalence of serum 25(OH)D deficiency in the present study, whether the findings could be extrapolated to serum 25(OH)D sufficient subjects needs to be investigated. Despite its limitations, the present study has some strengths, such as the inclusion of a wide range of ages of Japanese male and female subjects, and the objective measures of physical activity.

To our knowledge, our study is the first to consider the confounding effect of MVPA in the context of the relationship between 25(OH)D and inflammatory cytokine levels.

## 5. Conclusions

In conclusion, the prevalence of vitamin D deficiency in healthy Japanese adults is very high. Serum 25(OH)D concentrations are positively associated with IL-17 concentrations, but not IFN-γ or IL-6 concentrations, in healthy Japanese adults. This association is independent of physical activity.
